# A Copy Number Variant at the *KITLG* Locus Likely Confers Risk for Canine Squamous Cell Carcinoma of the Digit

**DOI:** 10.1371/journal.pgen.1003409

**Published:** 2013-03-28

**Authors:** Danielle M. Karyadi, Eric Karlins, Brennan Decker, Bridgett M. vonHoldt, Gretchen Carpintero-Ramirez, Heidi G. Parker, Robert K. Wayne, Elaine A. Ostrander

**Affiliations:** 1 National Human Genome Research Institute, National Institutes of Health, Bethesda, Maryland, United States of America; 2 Department of Ecology and Evolutionary Biology, University of California Los Angeles, Los Angeles, California, United States of America; University of Washington, United States of America

## Abstract

The domestic dog is a robust model for studying the genetics of complex disease susceptibility. The strategies used to develop and propagate modern breeds have resulted in an elevated risk for specific diseases in particular breeds. One example is that of Standard Poodles (STPOs), who have increased risk for squamous cell carcinoma of the digit (SCCD), a locally aggressive cancer that causes lytic bone lesions, sometimes with multiple toe recurrence. However, only STPOs of dark coat color are at high risk; light colored STPOs are almost entirely unaffected, suggesting that interactions between multiple pathways are necessary for oncogenesis. We performed a genome-wide association study (GWAS) on STPOs, comparing 31 SCCD cases to 34 unrelated black STPO controls. The peak SNP on canine chromosome 15 was statistically significant at the genome-wide level (P_raw_ = 1.60×10^−7^; P_genome_ = 0.0066). Additional mapping resolved the region to the *KIT Ligand* (*KITLG*) locus. Comparison of STPO cases to other at-risk breeds narrowed the locus to a 144.9-Kb region. Haplotype mapping among 84 STPO cases identified a minimal region of 28.3 Kb. A copy number variant (CNV) containing predicted enhancer elements was found to be strongly associated with SCCD in STPOs (P = 1.72×10^−8^). Light colored STPOs carry the CNV risk alleles at the same frequency as black STPOs, but are not susceptible to SCCD. A GWAS comparing 24 black and 24 light colored STPOs highlighted only the *MC1R* locus as significantly different between the two datasets, suggesting that a compensatory mutation within the *MC1R* locus likely protects light colored STPOs from disease. Our findings highlight a role for *KITLG* in SCCD susceptibility, as well as demonstrate that interactions between the *KITLG* and *MC1R* loci are potentially required for SCCD oncogenesis. These findings highlight how studies of breed-limited diseases are useful for disentangling multigene disorders.

## Author Summary

Domesticated dogs offer a unique mechanism for disentangling complex genetic traits, such as cancer. Over 300 breeds exist worldwide, each selected for particular morphologic and behavioral traits. Unfortunately the breeding programs used to generate such diversity are associated with breed-specific increase in disease. Squamous cell carcinoma of the digit (SCCD) is a locally aggressive cancer that causes lytic bone lesions and, occasionally, death. Among the breeds with the highest risk is the Standard Poodle (STPO), where the disease is found only in dark-coated dogs. We show that the *KITLG* locus is highly associated with SCCD and that a 5.7-Kb copy number variant is likely causative for the disease when in an expanded form. Interestingly, light-colored STPO carry the putative causal variant at the same frequency as black STPOs, but are protected from SCCD. We show this is likely due to a compensatory mutation in the well-known coat color locus, *MC1R*. This work demonstrates the utility of dog breeds for understanding the genetic causes of complex diseases of interest to both human and animal health.

## Introduction

Each of the approximately 300 domestic dog breeds recognized world-wide has undergone strong phenotypic selection for specific behavioral and morphologic traits. One consequence of the breeding programs used to propagate lineages with such strong phenotypic homogeneity is the increased incidence of diseases, including cancer. Indeed, cancer is the leading cause of disease-associated death in dogs [1], [2], with 23% of all dogs and 45% of dogs older than 10 years dying of cancer. Multiple breeds are at an elevated risk for specific cancers, indicating a likely genetic predisposition (reviewed in [3], [4], [5]). Dogs are diagnosed with nearly all of the same cancers as humans [6], and the underlying pathology and treatment response are typically the same as for humans [7], suggesting that canine cancer genetic studies are a useful way to advance our understanding of human disease [3], [8], [9].

Typically, for any given cancer, the number of deleterious alleles segregating in a single dog breed is likely to be limited, as dog fanciers employ closed breeding programs to develop breeds with specific phenotypic traits [5], [10], [11]. As a result, cancer gene mapping in dogs presents a mechanism to circumvent the small families, outbred population structure and locus heterogeneity that continually plague human cancer gene mapping [3], [4]. Applying the canine model to cancer gene mapping is particularly useful when multiple closely related breeds are at an increased risk for the same form of the disease [12], [13], [14], [15], as this often indicates that the breeds in question may share a common founder mutation. This is particularly applicable to the problem of cancer, where results from genome-wide association studies (GWAS) in humans indicate that noncoding variants are expected to contribute significantly to disease susceptibility [16].

Squamous cell carcinoma of the digit (SCCD) is the most frequently occurring cutaneous squamous cell carcinoma (SCC) in dogs, making up 60% of all SCCs of the skin [17], [18]. It is the most common malignant nail bed tumor, comprising 44.4% of reported cases [19], [20], [21], [22]. SCCD is a locally aggressive cancer that causes bone lysis in approximately 80% of cases [19], [20], [21]. Tumors can develop in multiple digits, typically in breeds at the highest genetic risk [17], [21], [22], [23]. The disease is considerably more aggressive than other cutaneous SCCs, with 19.2% of reported cases progressing to metastatic disease [19], [20], [21], [22], [24].

**Figure 1:**
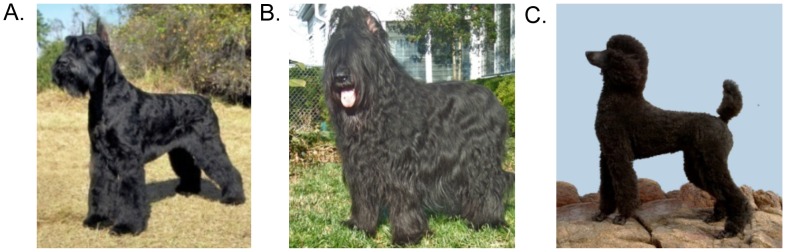
Multiple breeds have increased risk of SCCD. Photos of three at-risk breeds evaluated in this study are shown. A) Giant Schnauzer. B) Briard. C) Standard Poodle.

Multiple breeds have increased or decreased risk of SCCD compared to mixed breed dogs [24], [25]. The five breeds with the highest risk of SCCD include Giant Schnauzers (Odds Ratio (OR) = 22.7, 95% Confidence Interval (C.I.) = 16.0–32.3), Gordon Setter (OR = 11.1, 95% C.I. = 7.5–16.3), Briard (OR = 10.4, 95% C.I. 5.5–19.8), Kerry Blue Terrier (OR = 7.7, 95% C.I. = 4.8–12.2) and Standard Poodles (OR = 5.9, 95% C.I. 4.8–7.2) [25]. By comparison, three breeds with reduced risk are the Beagle (OR = 0.1, 95% C.I. = 0.03–0.30), Collie (OR = 0.16, 95% C.I. = 0.04–0.65) and Boxer (OR = 0.23, 95% C.I. = 0.13–0.43) [25]. In this study, we evaluated three increased risk breeds, Standard Poodles (STPOs), Giant Schnauzers and Briards (Figure 1). Phylogenetically, all three breeds belong to the modern group of dogs developed mostly in Europe, but are not closely related as none of them appear together within a single cluster or group [13], [26].

One of the most interesting aspects of SCCD is its profoundly strong association with dark coat colors in a subset of breeds [19], [20], [21], [22]. The most striking example is that of the very popular STPO where black dogs are at high risk for SCCD, but light colored dogs, including white and cream are, to our knowledge, unaffected. The association of the disease with particular STPO coat colors suggests that studies of SCCD might be informative for both identifying a cancer susceptibility allele as well as for elucidating additional complex gene or pathway interactions involved in the susceptibility process.

## Results

### STPO Genome-Wide Association Study

**Figure 2:**
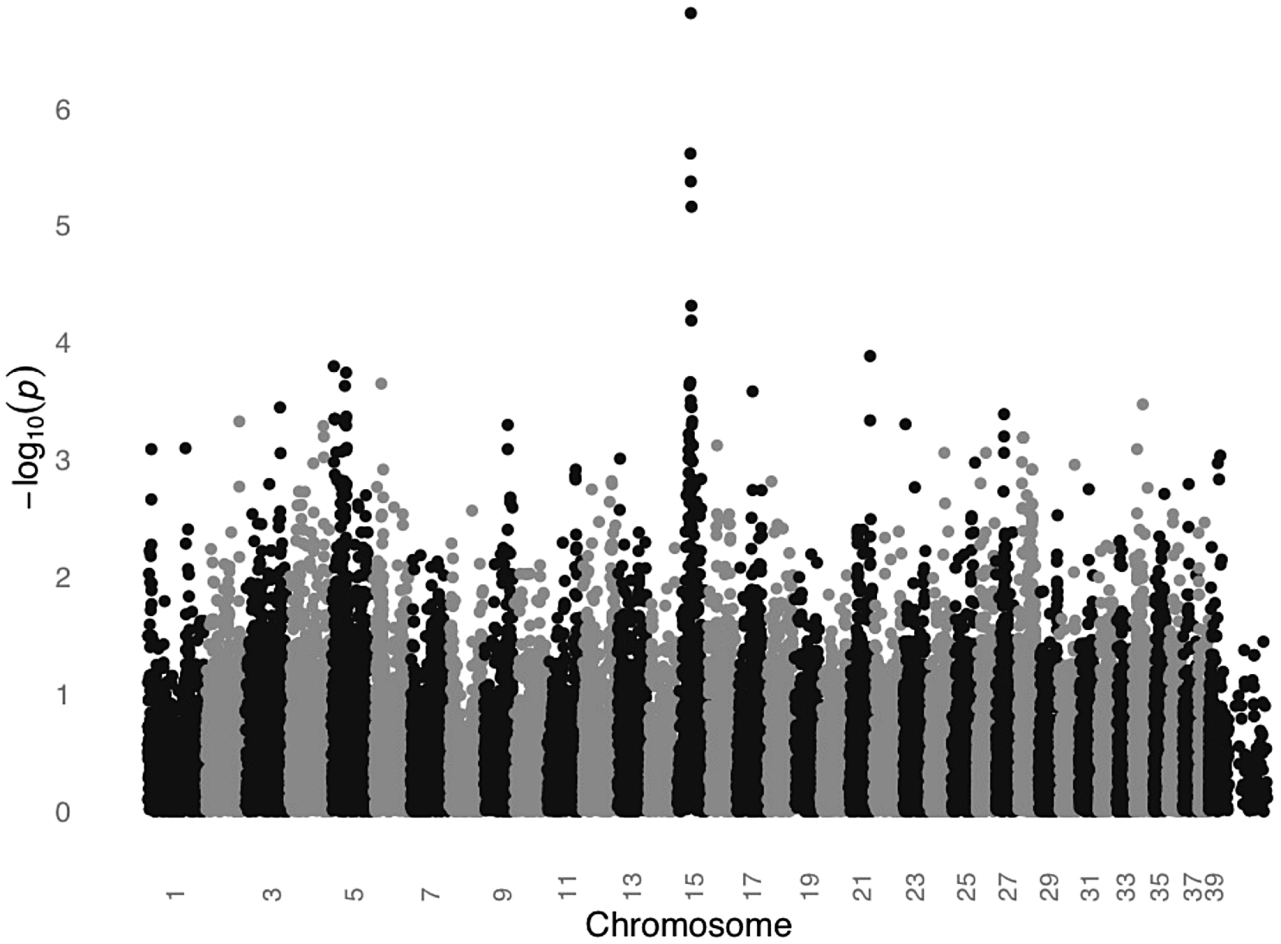
GWAS for SCCD in STPOs identifies a significant locus on CFA15. The GWAS compared 31 cases versus 34 controls at 36,897 SNPs. Values on the Y-axis represent the negative log of the uncorrected P value for association of each SNP with the disease phenotype from PLINK. The X-axis indicates the chromosome position in order from the top of CFA1 to the end of CFAX, which is labeled CFA39.

We conducted a GWAS for SCCD in STPOs using DNA from 31 cases and 34 controls (Figure 2). All cases had biopsy confirmation of SCCD and dark coats (30 black and one “blue”, which is a dilution of black). All controls had black coats, were over the age of eight and were unrelated to one another at the grandparent level. Analysis of 36,897 SNPs revealed a single statistically significant association on canine chromosome 15 (CFA15). The six most strongly associated SNPs (P_raw_ = 6.51×10^−5^ to 1.60×10^−7^) were contiguous on CFA15 and the peak SNP, CFA15:32,383,555 (in the CanFam2 assembly), was statistically significant at the genome-wide level (P_genome_ = 0.0066, based on 100,000 permutations). The risk-associated allele was present in 90.3% cases at the peak SNP, and 51.6% of cases were homozygous for the risk-associated allele (Table 1). By comparison, none of the controls were homozygous for the risk-associated allele, and 50% were heterozygous (Table 1).

**Table 1:** Association between SCCD risk and the peak GWAS SNP, CFA15:32,383,555, in STPO cases and controls.

		CFA15:32,383,555 Genotypes			P value	
	n	TT	TA	AA	P_raw_	P_genome_ a
Original STPO Cases	31	3	12	16	1.6×10^−7^	0.0066
Original STPO Controls	34	17	17	0		

a 100,000 permutations using PLINK Max(T).

### Recombination and Association Analyses

In order to refine the initial association peak further, both recombination mapping and an additional association analysis were performed. Both analyses utilized data from the resequencing of 525 amplicons in 38 cases and 30 controls. The cases represented the original 31 STPO cases plus seven additional cases that were enrolled following completion of the initial GWAS. The controls were the same as those in the initial GWAS, minus four with insufficient DNA for follow-up analyses. All 38 cases and 30 controls were resequenced around the peak SNP. Given that several cases did not carry the risk-associated allele at CFA15:32,383,555, we sought to capture variants in as many cases and controls as possible to assemble an optimally detailed haplotype structure across the region.

The 525 amplicons were spaced, on average, every one Kb within a 1.2 Mb region surrounding CFA15:32,383,555 (CFA15:31,900,000–33,100,000) in order to ensure that the boundaries of the recombination intervals and limits of the significant association signal were identified. The amplicons were also designed to resequence the exons of the six genes in the region. Following the resequencing, 862 variants, including SNPs and indels, were identified, for a median spacing of 370 bp. A total of 658 of 862 variants had a minor allele frequency >10%, generating a median spacing of 368 bp.

**Figure 3:**
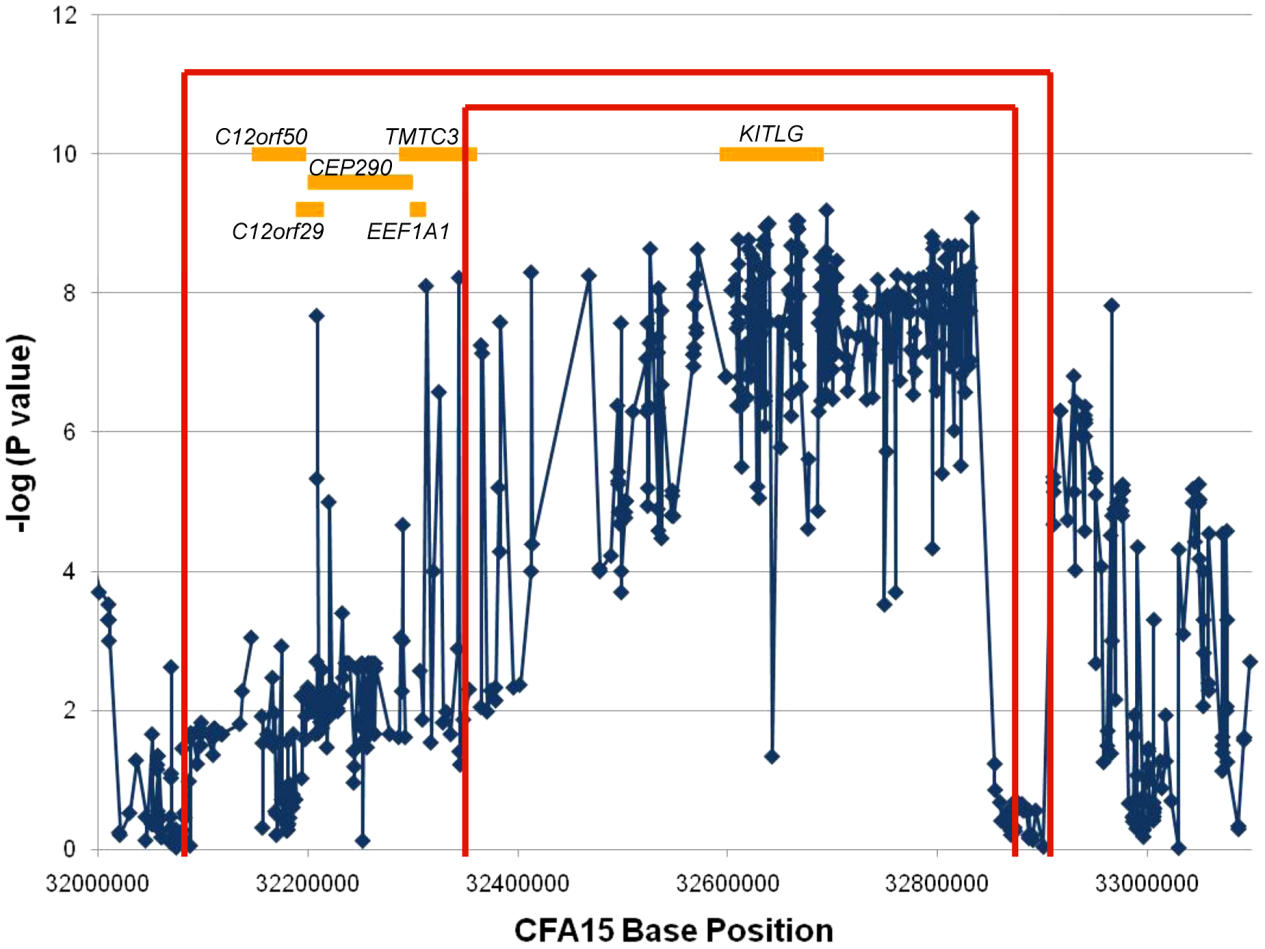
Recombination and association analyses resolve the region to the *KITLG* locus. The association analysis results for the 658 SNPs with minor allele frequencies >10% are plotted as the negative log of the uncorrected P value. The X-axis is the base position along CFA15. The results of the recombination analysis are shown as the red brackets with the inner and outer red brackets indicating the one and three recombination intervals respectively. The orange boxes are the known or predicted genes in the region, as labeled.

The recombination mapping highlighted regions where individual cases no longer shared at least one copy of the STPO risk-associated haplotype. For the one recombinant interval, the borders are defined by the position for which there is one individual case that no longer shares the putative risk associated haplotype. One individual may define the centromeric side of the region of interest and a different individual the telomeric border. The more conservative and standard approach is to define the borders of the region by the position where three individuals per border no longer share the region of interest. While this generally creates a larger region to analyze, it provides greater assurance that the region contains the mutation of interest since using one individual can create false positives if, for instance, that individual was misdiagnosed or represents a phenocopy. We utilized all 862 variants to determine the one and three recombination intervals. Since the analysis focused on finding where cases stop sharing the risk-associated alleles or haplotypes, only the 35 cases that shared at least one copy of the risk-associated allele at the peak SNP were included in the analysis. We evaluated one variant at a time and a recombination event was identified if a case no longer shared at least one copy of the STPO case major allele for a particular variant, and if this case continued to no longer share at least one copy of the STPO case major allele or haplotype for ≥one Kb. Centromeric to the peak SNP, the first recombination event was at 32,347,048 bp in one case and evidence for the third recombination event occurred at 32,088,047 bp in two additional cases (Figure 3). The first and third recombination events telomeric to the peak SNP were at 32,873,675 bp in one case and 32,901,086 bp in two additional cases, respectively. Thus, from this analysis, we defined the one recombination interval as the 526.6 Kb locus from CFA15:32,347,048–32,873,675 and the three recombination interval as the 813.0 Kb locus from CFA15:32,088,047–32,901,086 (Figure 3).

An additional association analysis was performed to highlight the region of strongest association to the disease. We compared data from all 38 cases to that obtained from the 30 controls. Figure 3 shows the association results for the 658 variants with minor allele frequencies >10%. All but three of the 220 variants that associated strongly with SCCD (P values<1.0×10^−7^) were within a single 520.1 Kb locus that spanned CFA15:32,312,908–32,832,982 (Figure 3), which corresponded closely to the 526.6 Kb one recombination interval. This region was observed to contain an excellent candidate oncogene, *KITLG*, whose exons are located entirely within the locus of association.

With such an obvious candidate gene at the SCCD locus, we intensified our efforts to obtain better coverage of the *KITLG* region. We eventually resequenced 100% of the exons, 89.7% of the introns, and 79.3% of the 10 Kb region upstream of the first exon (Figure S1A). We examined the data for a causal variant, defined as a variant which was present in all STPO cases and for which the risk allele frequency differed significantly between cases and controls. However, none of the variants identified in the region met these criteria. In addition, no new variant demonstrated a stronger association with the disease than those we had previously identified (Figure 3).

### Interbreed Haplotype Analysis

Since the STPO one and three recombination intervals are large, spanning 526.6 Kb and 813.0 Kb, respectively, we conducted an interbreed haplotype analysis to reduce the region of interest. We compared data from the 38 STPO cases with data from affected dogs of two other at-risk breeds: Giant Schnauzers (n = 28) and Briards (n = 11). All together, 536 variants with a median spacing of 472 bp were available for the STPO and Giant Schnauzer analysis, 821 variants with a median spacing 375 bp were evaluated for the STPO and Briard case comparison. Utilizing these variants, we scanned the STPO three recombination interval for the largest region where all Giant Schnauzer or Briard cases had at least one copy of the same haplotype as the majority of STPO cases.

**Figure 4:**
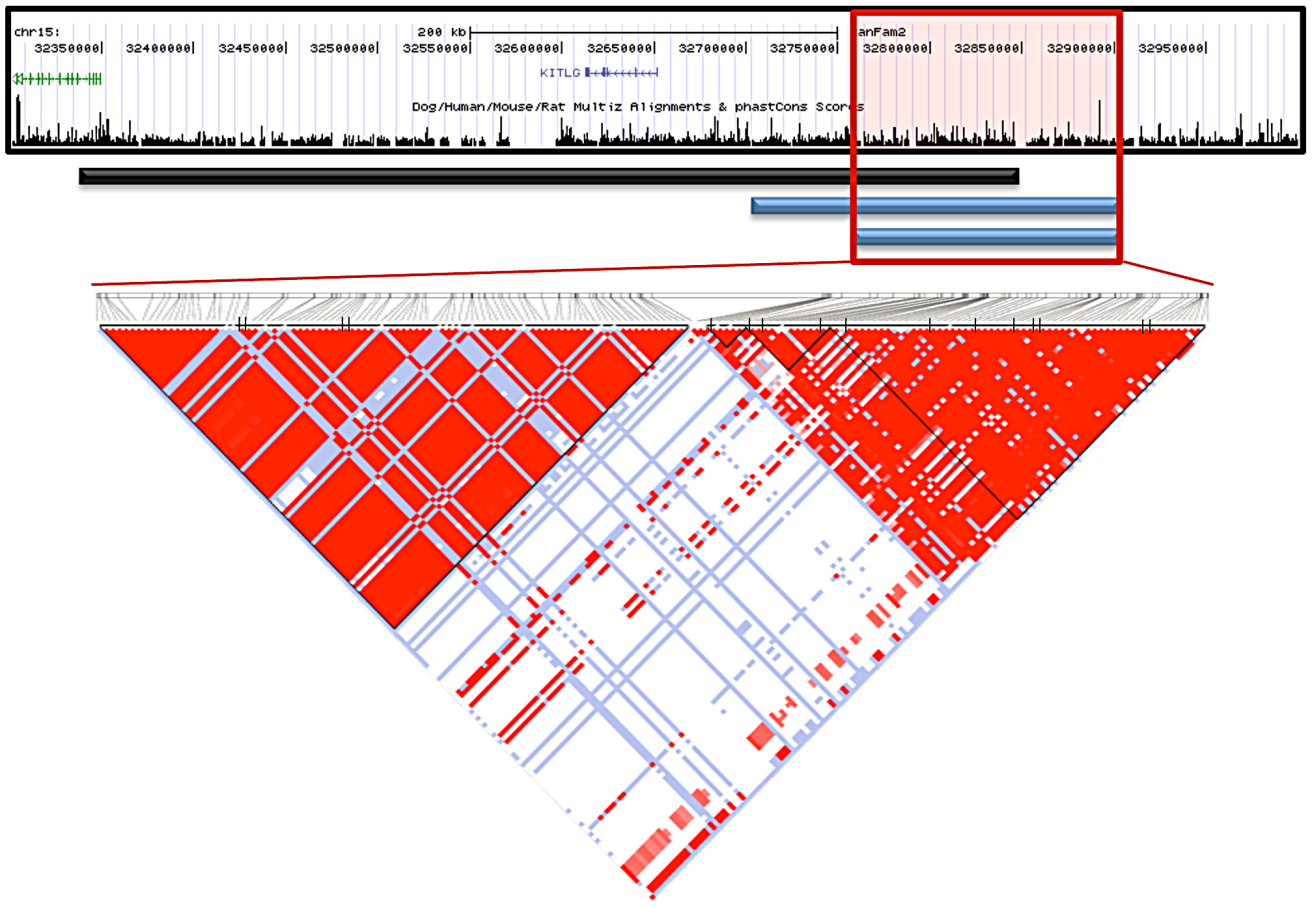
Interbreed haplotype analysis refines the SCCD locus to 144.9 Kb. The black bar represents the STPO one recombination region. The blue bars indicate the results of the interbreed haplotype analyses comparing STPOs with Giant Schnauzers (top blue bar) and Briards (bottom blue bar). The red box outlines the 144.9 Kb consensus region. The triangle plot displays the LD patterns in STPO cases (n = 38) and controls (n = 30) for the 186 variants within the region. The black vertical lines within the triangle plot indicate the locations of the tagging SNPs or in/dels within each block.

The Giant Schnauzers had only one region greater than 50 Kb where all cases carried the same haplotype as the majority of STPO cases. This region was 75.1 Kb in length, from CFA15:32,832,982–32,908,071, and the shared haplotype was present in 34 of 38 STPO cases. There were, however, four discordant SNPs in the adjacent region in either the Giant Schnauzer and/or STPO cases (CFA15:32,724,674, CFA15:32,749,603, CFA15:32,795,285 and CFA15:32,832,982) that likely arose on the risk haplotype after the causal variant. Acting conservatively we excluded these four SNPs from consideration, thus expanding the provisional region of interest, which we defined as that shared between the Giant Schnauzer and STPO cases, from 75.1 Kb to 207.8 Kb (CFA15:32,700,300–32,908,071; Figure 4).

The results of the interbreed haplotype analysis in the Briard cases were very similar to that of the Giant Schnauzer, although the Briards reduced the region of interest still further. Briards had only one region greater than 40 Kb for which all cases shared a haplotype with the majority of STPO cases, and it was the same as the initial Giant Schnauzer region (75.1 Kb from CFA15:32,832,982–32,908,071). The Briard cases were homozygous for the major STPO case haplotype, with the homozygosity extending to create a shared haplotype of 144.9 Kb between the Briard and STPO cases (CFA15:32,763,151–32,908,071; Figure 4), after discarding two of the discordant SNPs, described above, that also disrupted the Giant Schnauzer haplotype (CFA15:32,795,285 and CFA15:32,832,982). In summary, the largest overlapping region between STPO, Giant Schnauzer and Briard cases was the 144.9 Kb region that extends from CFA15:32,763,151 to 32,908,071.

We investigated the 144.9 Kb overlapping region in STPO cases and controls, and determined the linkage disequilibrium (LD) patterns of 186 STPO variants within the region using Haploview [27]. Two major LD patterns were present (Figure 4). The first had only one predicted LD block, which we termed block A. The second was comprised of three LD blocks, termed B, C and D, which were not very polymorphic in STPOs. The haplotype within block A segregated disproportionately with disease in the 38 STPO cases compared to the 30 controls (P = 1.67×10^−8^), and initially appeared promising as the location of the causal variant. The four SNPs that tag block A were therefore genotyped in all STPO, Giant Schnauzer and Briard cases and controls, including 46 additional STPO cases (84 total STPO cases). The number of dogs with zero, one or two chromosomes containing the risk-associated haplotype are indicated in Table 2. Importantly, not all STPO cases carried the block A risk-associated haplotype. Six of 84 did not, indicating that while variant(s) within block A were likely closely linked to the causal variant, they did not fully explain the disease in STPOs.

**Table 2:** Association between SCCD risk and the LD block A risk-associated haplotype in the STPO, Briard, and Giant Schnauzer cases and controls.

		Number of LD Block ARisk-associated Haplotypes			Frequency (%)	P Value^a^	OR (95% CI)^b^
	n	0	1	2			
All STPO Cases	84	6	32	46	73.8		
Original STPO Cases	31	3	12	16	71.0	5.73×10^−7^	5.9 (4.8–7.2)
Original STPO Controls	34	17	17	0	26.5		
Briard Cases	11	0	0	11	100	0.009	10.4(5.5–19.8)
Briard Controls	18	1	8	9	72.2		
Giant Schnauzer Cases	28	0	0	28	100	0.317	22.7(16–32.3)
Giant Schnauzer Controls	13	0	1	12	96.2		

a P values comparing original STPO cases and control, Briard cases and control or Giant Schnauzer cases and controls.

b Odds ratio (OR) and 95% confidence interval (CI) for SCCD in the breeds [25].

We noted, however, that the block A risk-associated haplotype was homozygous in all 28 Giant Schnauzer cases and nearly all of the unrelated Giant Schnauzer controls (12 out of 13; 96.2%), indicating that Giant Schnauzers are nearly fixed for this haplotype. Since the block A risk-associated haplotype was found to be closely linked to the causal variant in STPOs, the high frequency of the block A risk-associated haplotype in Giant Schnauzers hints at why they are at the highest risk for the disease (OR, 22.7; CI, 16–32.3) [25]. By comparison, 72.2% of Briard controls carried the STPO block A risk-associated haplotype, which was between the haplotype frequency in STPO (26.5%) and Giant Schnauzer (96.2%) controls, and was consistent with the disease risk observed in Briards (OR, 10.4; CI, 5.5–19.8) [25].

Since the variants identified thus far did not explain the disease in all STPO cases, we completed the resequencing of the 144.9 Kb region using tiled primers (Figure S1B). In the end, 142,938 bp or 98.6% of the region was completely resequenced in STPO cases and controls. The remaining 1,982 bp represented regions that were difficult to sequence using Sanger sequencing, including long stretches of homopolymers and other repeats. In the 144.9 Kb region, 36 additional variants that segregate with the disease were identified for a total of 114 disease-associated variants. However, no causal variant candidates that met our specified criteria of occurring in all STPO cases and being significantly associated with disease were identified.

**Figure 5:**
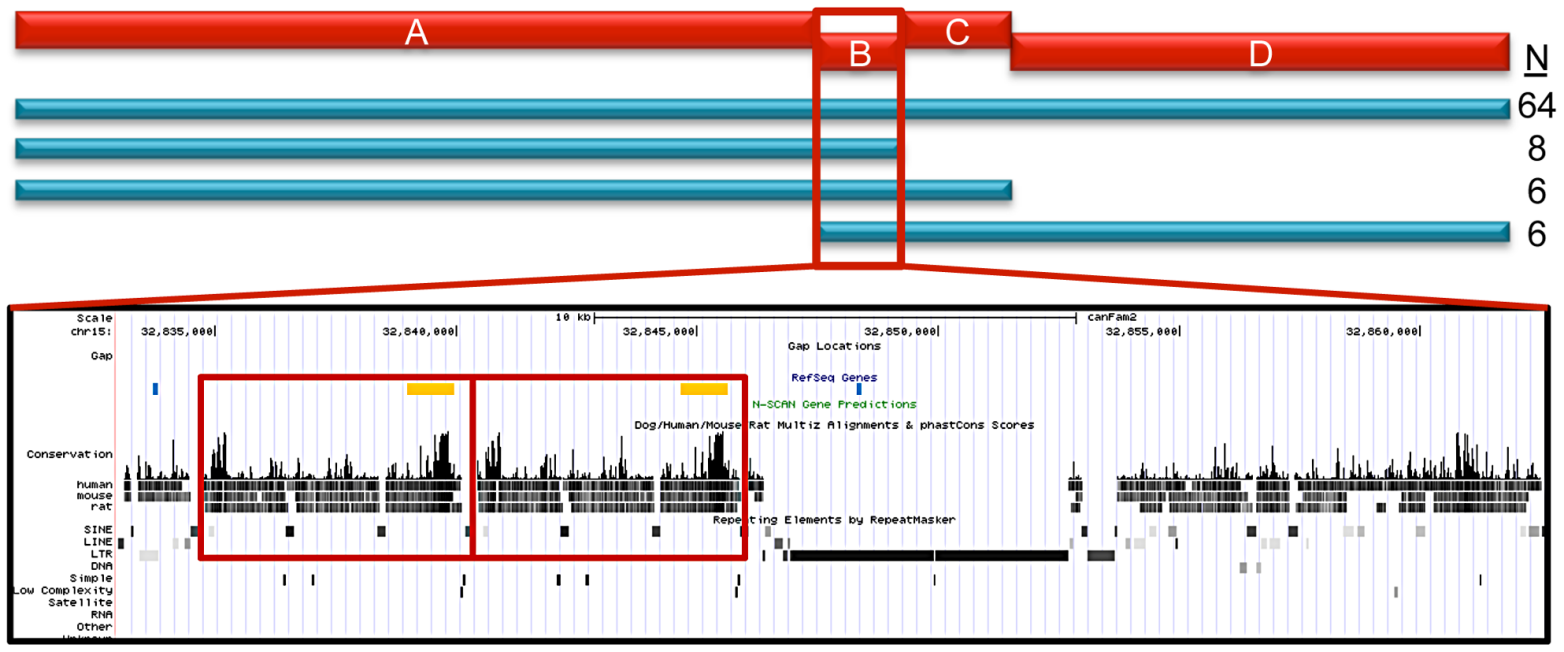
STPO cases narrow the region to 28.3 Kb. The red bars represent the four LD blocks in the 144.9 Kb region, termed A through D. The haplotype mapping results derived from 84 STPO cases are indicated by the blue bars, with the number of cases indicated on the right. The majority of STPO cases (n = 64) share at least one copy of the same haplotype within blocks A, B, C and D. Eight cases share in LD blocks A and B, six cases share in LD blocks A, B and C, and another six cases share in LD blocks B, C and D. The conservation and repeat elements in the reference genome for the 28.3 Kb from the end of block A to the beginning of block C are shown. The red boxes indicate the two copies of the 5.7 Kb element. Within the RefSeq Genes track, the blue lines are the PstI sites surrounding the CNV and the orange boxes are the locations of the probe used for the Southern blot.

We next performed haplotype mapping with tagging variants across the entire 144.9 Kb region in all 84 STPO cases (Figure 5) to identify regions to prioritize in a search for large insertions, deletions or copy number variants (CNVs). The majority of STPO cases (n = 64) shared at least one copy of the same haplotype within LD blocks A, B, C and D. The remaining 20 STPO cases shared at least one copy of the major STPO case haplotype in only two or three of the LD blocks, as indicated by the blue bars in Figure 5. Specifically, eight cases shared in LD blocks A and B, six cases shared in LD blocks A, B and C, and another six cases shared in LD blocks B, C and D. Thus, the only LD block where all STPO cases shared at least one copy of the same STPO case haplotype is LD block B. Data from the 38 STPO cases which were resequenced across the 144.9 Kb haplotype indicated the same result. As such, we investigated the 28.3 Kb between the end of LD block A and the beginning of LD block C further.

### Identification of the Putative SCCD Causal Variant

The canine reference sequence [15] for the 28.3 Kb sub-region contains a tandem copy of a 5.7 Kb element in the Boxer (Figure 5), which is only found once in all other placental mammalian species for which there is finished genome sequence (n = 20; http://genome.ucsc.edu). Interestingly, the 5.7 Kb element is, in fact, between the end of block A and the beginning of block B making it immediately adjacent to the block A disease-associated haplotype, which we observed was in strong, but not perfect, LD with the putative causal variant. To test if this was the disease variant, we performed Southern blots using DNA from STPO cases and controls where we identified variation in the copy number of the 5.7 kb unit ranging from one to five copies (Figure S2).

After comparing STPO cases and controls, our data indicated that the expanded 5.7 Kb element is an excellent SCCD causal variant candidate. All cases with Southern blot data (n = 47) had at least one allele with ≥4 copies of the 5.7 Kb element (Table 3), which we termed the risk alleles and, as such, the expanded CNV was strongly associated with disease in STPO cases (P = 1.72×10^−8^). Specifically, 15 cases were heterozygous and 32 were homozygous for ≥4 copies. By comparison, in a set of 45 unrelated black STPO controls, 13 had no alleles with ≥4 copies, 24 were heterozygous and eight were homozygous for the risk alleles (Table 3). Importantly, six out of 84 cases did not carry the block A risk-associated haplotype. Of these six, we were able to obtain CNV genotype data on four. All four cases had at least one copy of the CNV risk allele with three being heterozygous and one homozygous. We reevaluated the 144.9 Kb resequencing data and confirmed that no other SNP or small insertion/deletion had the same segregation pattern as the CNV risk and non-risk alleles. Thus, our data indicated that the CNV is the best SCCD causal variant candidate as the expanded 5.7 Kb element explained the disease in the STPO better than any of the other risk-associated variants identified.

**Table 3:** Association between SCCD risk and the 5.7-Kb element CNV in the STPO cases and controls.

	Number of LD Block ARisk-associated Haplotypes				Number of 5.7 Kb Element CNV Risk Alleles^a^			
	0	1	2	P Value	0	1	2	P Value
All STPO Cases	4	18	25	3.06×10^−8^	0	15	32	1.72×10^−8^
Black STPO Controls	20	22	3		13	24	8	

a Risk alleles are four or five copies of the CNV.

Evidence that the expanded CNV is the putative SCCD causal variant was also consistent with data obtained from the other increased risk breeds. All nine genotyped Giant Schnauzer cases were homozygous for four copies of the 5.7 Kb element. In the Briard, three out of the four genotyped cases were homozygous for the CNV risk alleles. Specifically, two Briard cases were homozygous for four copies, one was a four/five heterozygote and one was homozygous for two copies. Therefore, overall only one case out of 60 (47 STPOs, nine Giant Schnauzers and four Briards) lacked the putative causal variant. We also genotyped the CNV in 36 dogs from six breeds at reduced risk for SCCD (n = 3 to 8 dogs per breed). The six breeds selected, which varied in terms of size and morphologic features, were the Basset Hound (n = 8; OR 0.27; CI 0.10–0.73), Boston Terrier (n = 3; OR 0.25; CI 0.06–1.01), Boxer (n = 6; OR 0.23; CI 0.13–0.43), Shetland Sheepdog (n = 8; OR 0.18; CI 0.07–0.42), Collie (n = 7; OR 0.16; CI 0.04–0.65), and Beagle (n = 4; OR 0.10; CI 0.03–0.30) [25]. Alleles containing either one or three copies of the CNV were the most common in this population (52.8% and 41.7%). The two copy allele was infrequent (4.2%). As expected, the four copy allele was rare (1.4%) found in only one dog and none of the dogs carried the five copy allele. Thus, the CNV risk alleles are rare in the aggregate reduced risk breeds tested especially when compared to the set of 45 black STPO controls (P = 3.77×10^−10^). As the four copy allele was discovered in one of three unrelated Boston Terriers, it would be interesting to determine the population frequency of the four copy allele specifically in this reduced risk breed. However, no additional unrelated Boston Terriers were available in our collection at this time. Although we note that a much larger collection would be needed to determine the prevalence of the CNV risk alleles in SCCD increased or reduced risk breeds other than STPOs and in dog breeds as a whole, our data clearly indicated a strong and unique association between the expanded 5.7 Kb CNV and SCCD in STPOs.

Data from the corresponding human genome sequence (Chr12:89,170,403–89,176,159 in build GRCh37) suggested that the 5.7 Kb sequence contains elements of an enhancer binding site (Figure S3). Thus, we hypothesized that an enhancer-mediated increase in *KITLG* transcription is likely key to the disease process although we cannot exclude the possibility that the expanded CNV affects another gene in the region.

### Genetic Interaction with the Coat Color Gene, *MC1R*

Finally, we wanted to determine why black STPOs are uniquely at risk for SCCD while light colored STPOs are not. Of the 84 SCCD STPOs enrolled in our study, which included all that came to our attention and met the eligibility criteria in terms of pathology and disease status, 82 had a black coat color, one was blue (dilute black), one was brown and none had a light coat color (white, cream, apricot or red), in spite of the fact that light colored STPOs comprise approximately 31.8% of the STPO population in the United States, as calculated from the Standard Poodle Database version 6.2 [28]. We first tested the range of CNV alleles in 26 unrelated light colored STPOs (Table 4). We observed that the frequency of the risk alleles (≥4 copies of the CNV) in light colored STPOs was similar to that observed in 45 unrelated black STPO controls (P = 0.81) as well as the 34 unrelated, unaffected young STPOs chosen without regard to coat color, that served as population controls (P = 0.77). Additionally, the surrounding haplotypes on which the CNV alleles occur in the light colored and young STPOs were the same as the ones observed in the black STPOs. This indicated that the light colored STPOs carry the putative SCCD causal variant at a similar frequency as the black STPOs, and since the light colored STPOs do not get SCCD, some other factor or factors must protect them from the disease.

**Table 4:** Frequency of the 5.7-Kb element CNV in light colored, black, and young STPOs.

	Number of 5.7 Kb Element CNV Risk Alleles^a^			Frequency (%)	P Value	*mc1r* −/− (%)
	0	1	2			
Light STPO Controls	10	10	6	42.3	—	100
Black STPO Controls	13	24	8	44.4	0.81	0
Young STPOs	15	11	8	39.7	0.77	26.5

a Risk alleles are four or five copies of the CNV.

We hypothesized that the protection might be from a compensatory mutation(s) located elsewhere in the genome. Towards this end, the *melanocortin 1 receptor* (*MC1R*), a frequently studied pigmentation and skin cancer susceptibility locus, is the obvious candidate, as it is well established that a homozygous *MC1R* R306X mutation causes light versus dark coat color in STPOs and many other dog breeds [29]. We performed a GWAS to test whether the *MC1R* locus is the only locus where allele frequencies differ significantly between black and light colored STPOs. If it is, the locus could reasonably be proposed as protecting light colored STPOs from SCCD.

**Figure 6:**
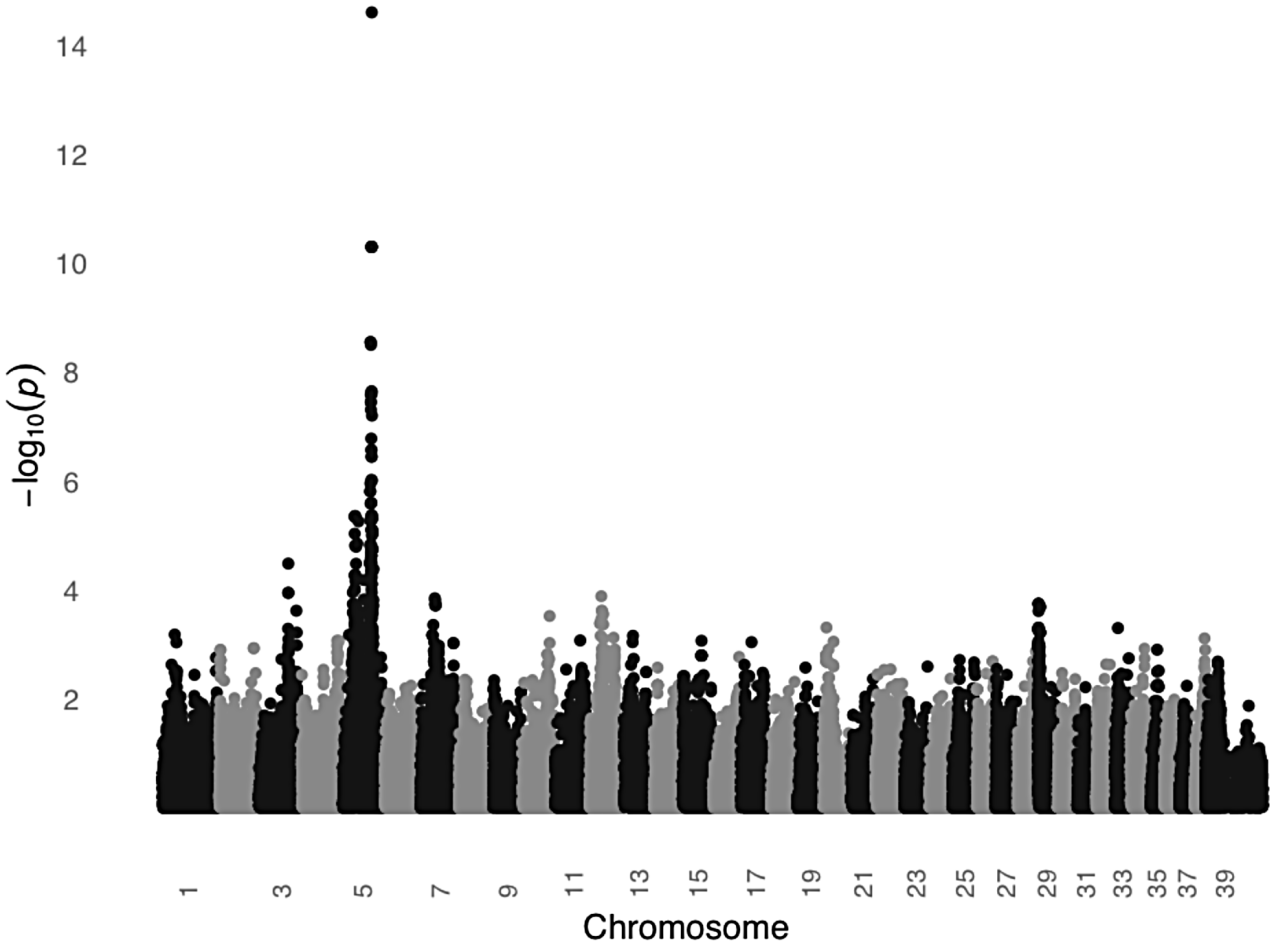
A significant association on CFA5 was identified in a GWAS comparing black and light colored STPOs. The GWAS compared 24 black and 24 light colored STPOs at 126,697 SNPs. Chromosome position is listed on the X-axis, and the negative log of the uncorrected P value of association of each SNP with the phenotype, as taken from EMMAX is indicated on the Y-axis.

In order to test the *MC1R* hypothesis, we performed a GWAS using DNA from 24 unrelated black STPO controls and 24 unrelated light colored STPO controls using the Illumina CanineHD BeadChip. Association analysis of 126,697 genome-wide SNPs revealed a single statistically significant result on canine chromosome 5 (CFA5, Figure 6). The 22 most strongly associated SNPs (P_raw_ = 3.58×10^−7^ to 2.52×10^−15^) were statistically significant at the genome-wide level after permutations and all were located on CFA5. The region of significant association extends from the peak SNP, CFA5:66,664,263 to CFA5:67,022,978 in the telomeric direction encompassing the entire *MC1R* locus. Importantly, no other locus was significantly different between black and light colored STPOs, indicating that the *MC1R* locus was the only candidate locus for the protection of light colored STPOs from SCCD.

We then genotyped the previously identified *MC1R* R306X mutation in the black and light colored STPOs we had sampled. The mutation segregated perfectly with coat color and had an identical segregation pattern as the peak GWAS SNP, CFA:66,664,263 (P = 2.52×10^−15^), indicating that the GWAS peak was likely tagging the previously identified mutation within *MC1R*. Although we cannot formally exclude the possibility that other variants in *MC1R* or nearby genes are in perfect LD with the peak GWAS SNP and the *MC1R* R306X mutation, our data supports the hypothesis that the *MC1R* R306X mutation is a good candidate for the protective variant, especially considering the mutation has functional consequences. The *MC1R* R306X mutation alters the protein and most likely acts as a loss-of-function allele. This is well supported by previously published studies of 833 dogs from 58 breeds in which the variant is consistently associated with light coat color [29], [30], [31], [32].

## Discussion

We demonstrate here the utility of the canine system for studying the genetics of complex traits such as cancer. Previous efforts to use the canine system for finding cancer genes have focused on either linkage studies of large single breed families, as was the case with canine cystadenocarcinoma [33], or have focused on diseases of single breeds, such as histiocytic sarcoma found in Bernese Mountain dogs [34]. While each study provided interesting and useful data, neither made extensive use of dog breed structure [13], [26], [35], which allows investigators to reasonably hypothesize that dogs with similar diseases who share recent common ancestors likely share both the disease haplotype and mutation [10], [13].

Indeed, in the case of SCCD, the fact that multiple breeds share the same haplotype at the disease locus was key in reducing a large region of association to 144.9 Kb, which could easily be interrogated by DNA sequencing. Prior to that, however, we performed a GWAS using DNA from 31 STPO cases and 34 unrelated STPO controls and identified a significant peak on CFA15 at CFA15:32,383,555. The fact that such a small number of individuals could be used for the GWAS was predicted by Lindblad-Toh [15], proven shortly thereafter [14], and is highlighted in a myriad of subsequent GWAS (for review see [8]). In the case of SCCD, after additional fine mapping in 38 STPO cases, we resolved the association peak to the *KITLG* locus. Comparison of STPO cases with cases from two other high-risk breeds refined the locus to 144.9 Kb. Haplotype analysis using 84 STPO cases narrowed the region to only 28.3 Kb, where we identified the putative SCCD causal variant as a 5.7 Kb CNV that is 183 Kb upstream of *KITLG*. Risk of SCCD in STPOs was strongly associated with the presence of the four copy or five copy allele of this CNV (P = 1.72×10^−8^), and all 47 STPO cases that we successfully tested carried at least one allele with ≥4 copies of the 5.7 Kb element. We found no STPO cases which lacked at least one copy of the risk allele.

Four studies have investigated CNVs genome-wide in dogs [36], [37], [38], [39]. Two of the four found evidence for the SCCD 5.7 Kb CNV region [36], [38]. In one study, all known canine CNVs were interrogated by array comparative genomic hybridization (aGCH) using DNA from 61 dogs representing 12 diverse breeds [38]. CNV loss was detected for several breeds and copy gains were found for one of the five Dachshunds and one of the six STPOs [38]. Interestingly, Dachshunds are another breed at increased risk for SCCD (OR = 2.2, 95% C.I. 1.6–3.0) [25].

Since the putative SCCD causal variant is a reiterated 5.7 Kb element located 183 Kb upstream of the primary gene, *KITLG*, it is interesting to hypothesize how the variant might modulate disease risk. Several studies have reported causal variant duplications in upstream regions leading to increased expression of nearby genes, including a study of hereditary mixed polyposis syndrome (HMPS) in humans and periodic fever syndrome in Chinese Shar-Pei dogs [40], [41]. HMPS is a Mendelian colorectal polyposis syndrome that results from an approximately 40 Kb duplication spanning the 3′ end of *SCG5* gene and the upstream region of the *GREM1* locus [41]. The duplication is associated with increased allele-specific expression of *GREM1* and not *SCG5* [41]. The HMPS duplication contains Encyclopedia of DNA Elements (ENCODE) predicted enhancer elements, some of which were shown to interact with the *GREM1* promoter and drive gene expression *in vitro* [41].

For the putative SCCD 5.7 Kb CNV causal variant, data from the corresponding human genome sequence (Chr12:89,170,403–89,176,159 in build GRCh37) suggests that it might also function to increase expression of nearby genes. The multiple species conservation site on the telomeric edge of the 5.7 Kb element contains elements of an enhancer binding site (Figure S3). Specifically, the ENCODE DNaseI Hypersensitivity analysis was positive in 47 out of the 148 cell lines tested including the only keratinocyte line assayed. The ENCODE Transcription Factor ChIP-seq analysis identified binding for four different transcription factors in cell lines that were derived from mammary epithelial tissue. Finally, the ENCODE and Broad Chromatin State Segmentation analysis by Hidden Markov Modeling predicted the presence of a strong enhancer binding site in both the normal epidermal keratinocytes and normal mammary epithelial cells. Therefore, one possible mechanism by which the risk alleles could affect disease susceptibility is that the additional copies of the 5.7 Kb element would create additional enhancer binding sites, which up-regulate transcription. As such, we hypothesize that the three, four and five copy alleles would each have a corresponding increase in expression and that the total number of copies also determine the level of transcription with, for example, individuals homozygous for the three copy allele having a lower expression level compared to individuals heterozygous for the three and four copy alleles.

If this mechanism of action for the 5.7 Kb CNV is validated, we further hypothesize that the CNV would affect SCCD risk in a dose-dependent manner, leading to an increase in disease penetrance for individuals carrying two versus one of the CNV risk alleles. Although our data is not a population-based sampling of STPOs, the proportion of cases among the total number of STPOs increases according to the number of CNV risk alleles (Table 3; zero risk alleles, 0%; one risk allele, 38.5%; two risk alleles, 80%), suggesting an increase in disease penetrance with two risk alleles compared to only one risk allele. At the same time, our data suggests that age-dependent penetrance or incomplete penetrance might also be involved to account for the small number of dogs that are homozygous for the risk allele and do not yet have the disease. Of course, we cannot formally exclude the possibility that a variant at another locus further modifies disease susceptibility. Finally, since our hypothesis indicates that the five copy allele would have higher expression compared to the four copy allele, it would be interesting to look at the correlation between genotype and phenotypes like age at onset or recurrence of SCCD once we have a large enough collection of STPO cases with the five copy allele. While functional studies would provide more definitive support for the 5.7 Kb CNV mechanism of action, the optimal experiment is difficult to perform in pet dogs, as expression studies would require difficult to obtain nail bed tissue from STPOs, with and without the putative causal variant CNV. Owners of both cases and, especially, controls are understandably reluctant to provide such tissue from their pets, as it would incur significant discomfort.

*KITLG* encodes the ligand for the tyrosine kinase receptor KIT. Together they are involved in multiple processes including melanocyte development and epidermal homeostasis and, as such, play a role in pigmentation. Specifically, *KITLG* has been associated with intensity of hair color pigmentation in humans [42], [43]. In stickleback fish, it is associated with skin pigmentation such that decreased *KITLG* expression reduces pigmentation [44]. When the *KITLG* locus was examined in humans, Miller et al. found strong signatures of selection in Europeans and East Asians and an association with skin pigmentation from admixture mapping in African Americans, suggesting that the *KITLG* locus contributes to human skin pigmentation as well [44].

Interestingly, like the human *KITLG* locus, the canine locus is also under strong selection. It is one of the top 20 loci with signatures for selection as assessed by *F_ST_* in two independent datasets [45], [46]. In the study of Boyko et al., which included 80 dog breeds and over 900 individuals, the *F_ST_* region at *KITLG* extends from CFA15:32,383,555–33,021,330, which starts at the original peak SNP of the SCCD GWAS and extends beyond the 5.7 Kb CNV [45]. In the study of Vaysse et al., the *F_ST_* region observed in 46 breeds is smaller, extending from CFA15:32,638,117–32,853,840, but it still overlaps the putative causal variant 5.7 Kb CNV [46]. Given the effect of KITLG on hair and skin pigmentation intensity in humans, the signature of selection at *KITLG* in dogs most likely represents breeders' attempts to propagate dogs of a certain color. Additional work is required to demonstrate if the SCCD 5.7 Kb CNV risk haplotypes specifically have signatures of selection within this locus. However, since the putative SCCD causal variant is well within a region under strong selection, it is intriguing to think that the SCCD susceptibility locus might be one of the first to demonstrate what has long been hypothesized for dogs that breeder-based trait selection can unknowingly lead to the entrapment of cancer causing alleles [5], [9], [10].

KITLG/KIT signaling has also been implicated in oncogenesis. The *KITLG* locus was initially identified as a cancer susceptibility locus for human testicular germ cell tumors in two independent GWASs, although the specific mutation and mechanism of action remains unknown [47], [48]. In addition, somatic activating *KIT* mutations are associated with several cancers, including human gastrointestinal stromal tumors and human melanomas (reviewed in [49], [50]). However, our study is the first to report the involvement of the *KITLG* locus in skin cancer susceptibility.

One interesting finding from our data is that the *MC1R* locus is the only candidate locus for the putative protection of light colored STPOs from SCCD. If a functionally active *MC1R* is proven to be required for SCCD susceptibility, we hypothesize that this is likely due to a necessary interaction of the MC1R pathway with the KITLG/KIT pathway to promote SCCD oncogenesis and/or that dark pigmentation is required within the nail bed. Although we cannot formally exclude the more distant possibility that the protection from SCCD is provided by a mutation in another gene or genetic element within the *MC1R* locus selective sweep, we believe that a loss-of-function mutation in *MC1R* is the most likely cause since both pathways are known to be involved in oncogenesis and there is previous evidence for multiple interactions between the two pathways. One such interaction involves signaling from MC1R, which can cause transactivation of the KIT receptor via Src tyrosine kinase [51]. Additionally, signaling from both KITLG and MC1R affects the MITF transcription factor, whose functions include cell cycle regulation and antiapoptotic signaling [52]. Indeed, MC1R pathway activity increases *MITF* expression and the KIT pathway phosphorylates MITF via the MAPK pathway to activate the protein [52]. Thus, both pathways could interact in SCCD oncogenesis via MITF. While the specifics of why functionally active MC1R signaling is required for SCCD oncogenesis remains elusive, our study identified a potential genetic interaction between the *KITLG* and *MC1R* loci such that mutations in the *MC1R* locus may be responsible for protecting dogs from KITLG-induced SCCD susceptibility.

One unanswered question from this study is how alteration of the KITLG/KIT and MC1R signaling pathways lead to SCCD, since both pathways are known to function within the melanocyte and not necessarily within the keratinocyte, which is the originating cell for SCCD. One theory for how the putative overexpression of *KITLG* can lead to SCCD is that the keratinocyte and melanocyte are adjacent cells in skin with well known paracrine activity between the cell types [53]. Indeed, KITLG is produced in the keratinocyte and released to simulate the melanocyte, which is how the KITLG/KIT pathway regulates important communication signals between the melanocytes and surrounding keratinocytes in the skin [53]. Therefore, it is possible that some other factor(s), perhaps one of the cell cycle or antiapoptotic factors downstream of MITF, that are produced in the melanocyte might promote oncogenesis in the keratinocyte.

If loss-of-function mutations in *MC1R* are proven to protect canines from SCCD, it may initially seem to be a surprising result, as loss-of-function *MC1R* variants are associated with increased incidence of skin cancer in humans. Consistently, a relationship between the *MC1R* loss-of-function ‘R’ alleles (D84E, R142H, R151C, I155T, R160W, and D294H) and risk of cutaneous basal cell carcinoma (OR 1.37–3.16), SCC (OR 1.99) and melanoma (OR 1.38–4.64) has been shown (reviewed in [54]). However, our study is not the only one to suggest that functionally active MC1R signaling may promote rather than protect against skin cancer incidence/progression. Rather, a study of melanoma in gray horses where increased wild-type MC1R signaling was shown to promote melanoma incidence was the first [55], and additional supporting data comes from studies of melanoma survival in humans [56]. Specifically, individuals who were homozygous for *MC1R* mutant alleles had a significantly lower risk of melanoma-specific death in a series of 3060 cases from Europe and the United States (HR, 0.78; 95% CI, 0.65–0.94), implying that a functional MC1R pathway promotes melanoma progression in humans. Both studies are consistent with our findings for SCCD in STPOs. A functionally active MC1R pathway can play a distinct role in oncogenesis unique from what has been proposed previously, i.e. functional MC1R signaling does not always protect against, but can actively promote cancer incidence and/or progression.

Our findings highlight the value of studying complex diseases in non-human systems such as the dog, where we have the ability to exploit breed-specific reduced genetic variability and interbreed relatedness to find genetic variants. Our studies of SCCD allowed us to not only identify a single cancer susceptibility causal variant candidate, but also a multiple locus interaction that would be difficult to uncover in a genetically diverse population. These discoveries, if confirmed in future analyses, not only allow us to better understand the interplay between two well-studied pathways, but provide additional evidence that the MC1R pathway can contribute to oncogenesis in multiple ways.

## Materials and Methods

### Ethics Statement and Sample Collection

All samples were collected from pet dogs after the owners provided informed consent. Study materials were approved by the Animal Care and Use Committees at the collection institutions. All procedures and materials were approved by the Animal Care and Use Committee of the National Human Genome Research Institute. DNA from blood was extracted using standard protocols. Saliva DNA was collected and extracted using the Oragene-Animal collection kit (DNA Genotek, Ontario, Canada).

SCCD cases were all confirmed with biopsy reports from veterinary pathologists. Controls are ≥8 years old at the time of the analysis with pedigree information and unrelated at the grandparent level. For the original STPO controls (n = 34), Briards (n = 18) and Giant Schnauzers (n = 13), dogs were considered controls if born before 2002 and unaffected. In the 5.7 Kb CNV analysis and black versus light colored STPO GWAS, the unrelated black STPO controls (n = 45) and light colored controls (n = 26) were born before 2005, while the young STPOs (n = 34) were born in or after 2005.

### GWAS Analyses

The first GWAS compared 31 STPO cases and 34 unrelated black STPO controls using the Affymetrix v2 Canine SNP Chip (Affymetrix, Santa Clara, CA). The BRLMM-P algorithm was used to genotype the SNPs. SNPs were removed from the analysis if greater than 10% of the data were missing, there were more than 60% heterozygous calls, or the minor allele frequency was <5%. The final dataset consisted of 36,897 SNPs.

The second GWAS compared 24 unrelated black STPO controls and 24 unrelated light colored STPO controls using the Illumina CanineHD BeadChip (Illumina, San Diego, CA). SNP genotypes were called using the Illumina Genome Studio software package. SNP clusters were evaluated if the call rate was <90%, the heterozygous excess was −1 to −0.7 or 0.5 to 1, and if the GenTrain score was <0.5. SNPs were removed from the analysis if the evaluated SNP clusters could not be improved or if the minor allele frequency was <5%. The final set consisted of 126,697 SNPs. The genotypes and phenotypes for both GWASs will be submitted to Gene Expression Omnibus (GEO).

Both datasets were analyzed for population stratification using principle components analysis. The principle components (PCs) were calculated in Eigenstrat [57] and Tracy-Widom statistics were utilized to determine if the PCs were statistically significant. For those deemed significant (TW p-value< = 0.05), ANOVA F-statistics were calculated within the assigned populations (either case/control for the first GWAS or black/light in the second GWAS) to determine if the PCs divided the population based on the phenotype of interest.

The SCCD case/control GWAS did not have evidence of population stratification by phenotype and was analyzed by calculating the allelic association (P_raw_) of each SNP with the disease using the statistical package PLINK v1.06 (http://pngu.mgh.harvard.edu/purcell/plink/) [58]. Correction for multiple testing was performed using 100,000 MaxT permutations in PLINK (P_genome_). The results of the MaxT permutations matched those obtained from Bonferroni correction at the 0.05 level.

The GWAS comparing black/light coat colored STPOs did have evidence for population stratification by phenotype and the allelic association (P_raw_) of each SNP with the phenotype was performed using the program EMMAX to correct for population structure [59]. To correct for false positive associations due to multiple testing, phenotypes were randomly permuted and association was repeated 1000 times. P_genome_ values were based on the number of permutations out of 1000 that produced an equal or lower result. The results of the EMMAX-based permutations also matched those obtained from Bonferroni correction at the 0.05 level.

### Sequencing

Over 1,268 amplicons were sequenced within the broad original genomic region (CFA15:30,280,088–35,714,394). Primers were designed using Primer3 v0.4.0 [60]. Primer sequences are available (Table S1). Amplification used standard PCR methods and sequencing was done using BigDye Terminator v3.1 on an ABI 3730xl DNA Analyzer (Applied Biosystems, Life Technologies, Grand Island, NY). Sequences were analyzed using the Phred/Phrap/Consed software packages [61], [62], [63] and SNPs were identified using Polyphred [64]. Typically, 38 STPO cases and 30 STPO controls were resequenced. However, for a small proportion of the *KITLG* introns, the *KITLG* upstream region and the 144.9 Kb overlapping region, a set of six STPO cases and four STPO controls were resequenced. The ten samples were selected to represent the haplotypes in the region. Of the cases, two were homozygous for the risk haplotype, two were heterozygous and two were homozygous for the non risk haplotype. For the controls, three were heterozygous for the risk haplotype and one was homozygous for the non risk haplotype. Genotypes for the variants identified are available (Table S1).

### Recombination and Association Fine-Mapping

Recombination mapping was performed with 862 variants identified from resequencing 38 STPO cases and 30 STPO controls in a 1.2 Mb region surrounding the initial peak (CFA15:31,900,000–33,100,000). Seven new cases were enrolled subsequent to the initial GWAS for a total of 38 STPO cases. We evaluated only the 35 cases which shared at least one copy of the risk-associated allele at the peak SNP, CFA15:32,383,555, for recombination mapping. In this analysis, a position was identified as the location of a recombination event if a case no longer shared at least one copy of the STPO case major allele (i.e. homozygous for the STPO case minor allele). Additionally, the case needed to continue to be homozygous for the STPO case minor allele for more than one Kb. We set this requirement in order to take the most conservative approach, and to only identify persistent changes in the risk-associated haplotype pattern. We note that within the entire 1.2 Mb region only four variants were excluded as recombination events since the change in genotype did not persist for more than one Kb. For two variants, the minor allele only occurred in a subset of dogs with the risk haplotype indicating that these variants are likely mutations that arose on the haplotype after the causal variant. The other two variants were only 611 bp apart and the STPO case minor allele is the STPO control major allele. In this case, the association with disease is equally as strong, either centromeric or telomeric of these variants. As such, these two variants most likely resulted from a series of unique crossover events in different generations on the risk haplotype where we cannot unambiguously determine that the causal variant is within the centromeric or telomeric section.

The additional association analysis compared all 38 STPO cases to 30 unrelated black STPO controls. Four controls were excluded since they did not have sufficient DNA. The allelic association was calculated for all 862 variants in a 1.2 Mb region surrounding the initial peak (CFA15:31,900,000–33,100,000). Since none of the variants with minor allele frequencies <10% were significant, the data was plotted with the 658 variants having minor allele frequencies >10%, for clarity (Figure 3). As with the GWASs, the allelic association of each variant with the disease phenotype was calculated using PLINK v1.06.

### Interbreed and STPO Haplotype Analyses

For the haplotype mapping comparing either Giant Schnauzer (n = 28) or Briard cases (n = 11) to the STPO cases (n = 38), we scanned the STPO three recombination interval (CFA15:32,088,047–32,901,086) for the largest region of haplotype sharing. In the previous resequencing effort, either the Giant Schnauzer or Briard cases were sequenced with the STPO cases and controls. Variants from this resequencing were included in the interbreed haplotype analysis along with additional variants, which were then genotyped in either breed to make sure that there was a variant, on average, every 1500 bp. First, we started at the centromeric edge of the interval, CFA15:32,088,047, and moved variant by variant through the interval. We identified variants where all Giant Schnauzer or Briard cases had at least one copy of the STPO case major allele (i.e. where no Giant Schnauzer or Briard cases were homozygous for the STPO case minor allele). We then calculated the size of these intervals and the haplotypes within each interval were determined. If the haplotype started within the three recombination region, we included the area until the end of the haplotype sharing with STPO cases. Finally, the regions of interest were intervals where the Giant Schnauzer or Briard cases share at least one copy of the same haplotype as the majority of STPO cases (>50%). Four SNPs, CFA15:32,724,674, CFA15:32,749,603, CFA15:32,795,285 and CFA15:32,832,982, assumed to have arisen on the haplotype after the mutation or as the result of a series of unique crossover events in separate generations, disrupted the haplotype sharing between STPO, Giant Schnauzer and/or Briard cases, and were removed from future analyses.

The LD block patterns in the 144.9 Kb overlapping region were determined with 186 variants in the STPO cases (n = 38) and controls (n = 30) using the Confidence Interval analysis in Haploview v4.1 [27]. Tagging variants (SNPs or indels) were selected to capture haplotypes predicted by Haploview. There were four SNPs to capture the six haplotypes in LD block A (CFA15:32,782,292 G/A; CFA15:32,782,334 A/G; CFA15:32,796,712 T/C; CFA15:32,796,907 C/A), with the risk-associated haplotype being G/A/T/C at these SNPs, respectively. One SNP captured the two haplotypes in LD block B (CFA15:32,854,334), three SNPs captured the four haplotypes in LD block C (CFA15:32,859,750, CFA15:32,862,724, CFA15:32,870,197), and eight SNPs and indels captured the nine haplotypes in LD block D (CFA15:32,871,555, CFA15:32,876,284, CFA15:32,880,662, CFA15:32,887,141, CFA15:32,888,465, CFA15:32,888,492, CFA15:32,898,733, CFA15:32,899,461).

### Southern Blot Analysis

The nonradioactive DIG Southern blot system (Roche Applied Science, Gilroy, CA) was used to analyze the 5.7 Kb CNV. Briefly, 1–2 µg genomic DNA was digested with the PstI enzyme for 3 hours at 37°C. High molecular weight DNA was necessary for the assay. Samples were run at 30 volts for 16 hours on a 0.6% agarose gel in TAE buffer. A control that was heterozygous for the three and four copy alleles was run on every Southern. The bands on the Southern blot were the following sizes after digestion: 8.2 Kb, 13.9 Kb, 19.6 Kb, 25.3 Kb and 31 Kb for the one, two, three, four and five copy alleles, respectively. A subset of samples were sufficiently degraded such that interpretable results were not obtained. All owners of STPO cases were recontacted to provide additional saliva samples. Of the 37 STPO cases without Southern results, 29 had already passed away. For the remaining eight, we were unable to collect a high quality saliva sample. Ultimately, out of the 84 STPO cases that were attempted, Southern blot data was available for 47 (55.6%). We experienced similar results for the STPOs of control age.

Blots were processed as specified by the DIG Southern blot system (Roche Applied Science) for hybridization targets ≥5 Kb. The probe was prepared using the PCR DIG Probe Synthesis kit (Roche Applied Science) with half DIG labeled dNTPs and half regular dNTPs in a two step PCR reaction (68°C annealing and extension temperatures) with the following primers, CTGATTCACATTTCCAAGGTGACAATGA and ACATGGCAGAGAAAGGCAACTAAGACCT. The DIG labeled probe was quantitated on a 1% agarose gel by comparing the probe intensity with the intensity of the Low Mass Ladder (Invitrogen, Carlsbad CA). The DIG Easy Hyb buffer (Roche Applied Science) was used for both the pre-hybridization and hybridization solutions. For hybridization, the probe concentration was 10 ng/ml. The low/high stringency washes and the DIG wash and block buffer set washes were performed according to the DIG protocol using CPD-star (Roche Applied Science). Blots were exposed to X-ray film for 30 minutes to two hours depending on the intensity of the signals.

## Supporting Information

Figure S1 Graphical representation of the sequence coverage across the *KITLG* gene and the 144.9 Kb interbreed haplotype analysis region. The UCSC Genome Browser display for the *KITLG* gene (A) and the 144.9 Kb interbreed haplotype analysis region (B). Multiple tracks are shown, including the RefSeq Genes predictions and the conservation tracks. The red bars indicate the basepairs successfully resequenced in STPOs.
(TIF)

Figure S2 Southern blot detecting the three, four and five copy alleles at the SCCD 5.7 Kb CNV element. A Southern blot of PstI digested genomic DNA is shown. The sizes for the three, four and five copy alleles are 19.6 Kb, 25.3 Kb and 31 Kb, respectively. The CNV genotypes are as indicated above each lane, with lanes 1 and 10 containing a high molecular weight ladder (L).
(TIF)

Figure S3 The human genome sequence that corresponds to the SCCD 5.7 Kb CNV contains enhancer element signatures. The UCSC Genome Browser display is of human chromosome 12 from 89,170,403 to 89,176,159 (build GRCh37). Multiple tracks are presented including the ENCODE DNaseI Hypersensitivity, ENCODE ChIP-seq and ENCODE/Broad Chromat in State Segmentation tracks.
(TIF)

Table S1 Primers, genotypes and alleles in the SCCD associated region. The Primers worksheet has all the primer sequences listed. The STPO_SCCD_Recomb_Assoc worksheet has the genotypes for the 862 variants utilized in the recombination mapping and association analysis. The GSCH_IHA worksheet has the genotypes for the 536 variants used in the interbreed haplotype analysis comparing Giant Schnauzer and STPO cases. The BRID_IHA worksheet contains the genotypes for the 821 variants utilized in the interbreed haplotype analysis evaluating Briards and STPO cases. The SCCD_114_Hap_DAV worksheet contains the 114 disease associated variants identified from the resequencing of the 144.9 Kb region.
(XLSX)
