# Supplementary figures and images for: A Copy Number Variant at the KITLG Locus Likely Confers Risk for Canine Squamous Cell Carcinoma of the Digit
Source: PLoS Genet. 2013 Mar 28;9(3):e1003409. doi: 10.1371/journal.pgen.1003409 (PMC3610924; doi:10.1371/journal.pgen.1003409)

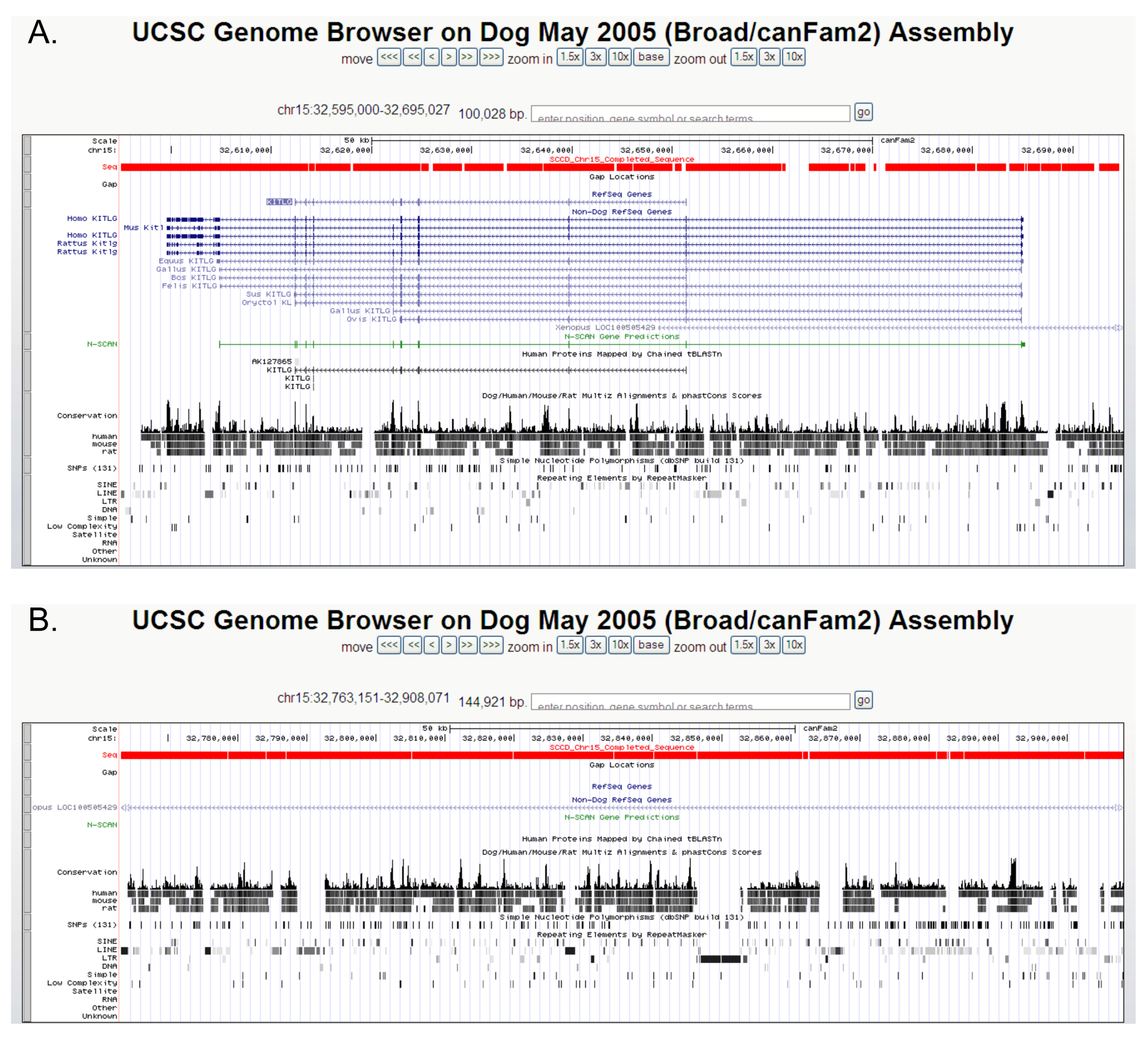

Supplement: Figure S1 — Graphical representation of the sequence coverage across the KITLG gene and the 144.9 Kb interbreed haplotype analysis region. The UCSC Genome Browser display for the KITLG gene (A) and the 144.9 Kb interbreed haplotype analysis region (B). Multiple tracks are shown, including the RefSeq Genes predictions and the conservation tracks. The red bars indicate the basepairs successfully resequenced in STPOs. (TIF) [file pgen.1003409.s001.tif]

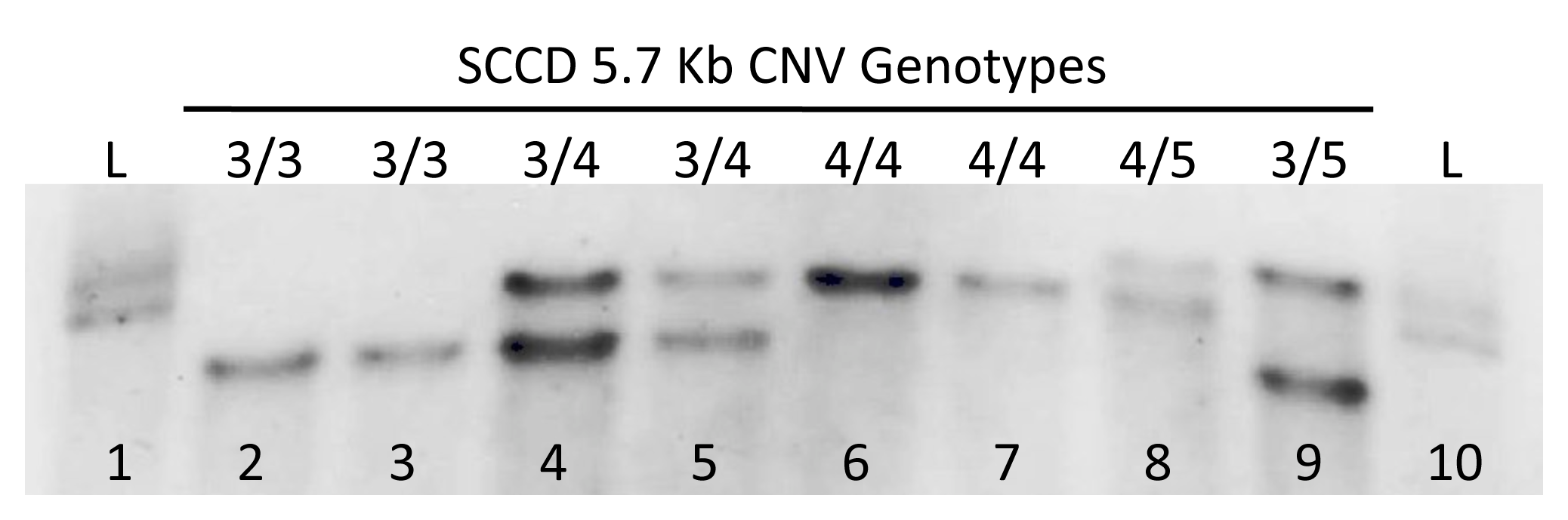

Supplement: Figure S2 — Southern blot detecting the three, four and five copy alleles at the SCCD 5.7 Kb CNV element. A Southern blot of PstI digested genomic DNA is shown. The sizes for the three, four and five copy alleles are 19.6 Kb, 25.3 Kb and 31 Kb, respectively. The CNV genotypes are as indicated above each lane, with lanes 1 and 10 containing a high molecular weight ladder (L). (TIF) [file pgen.1003409.s002.tif]

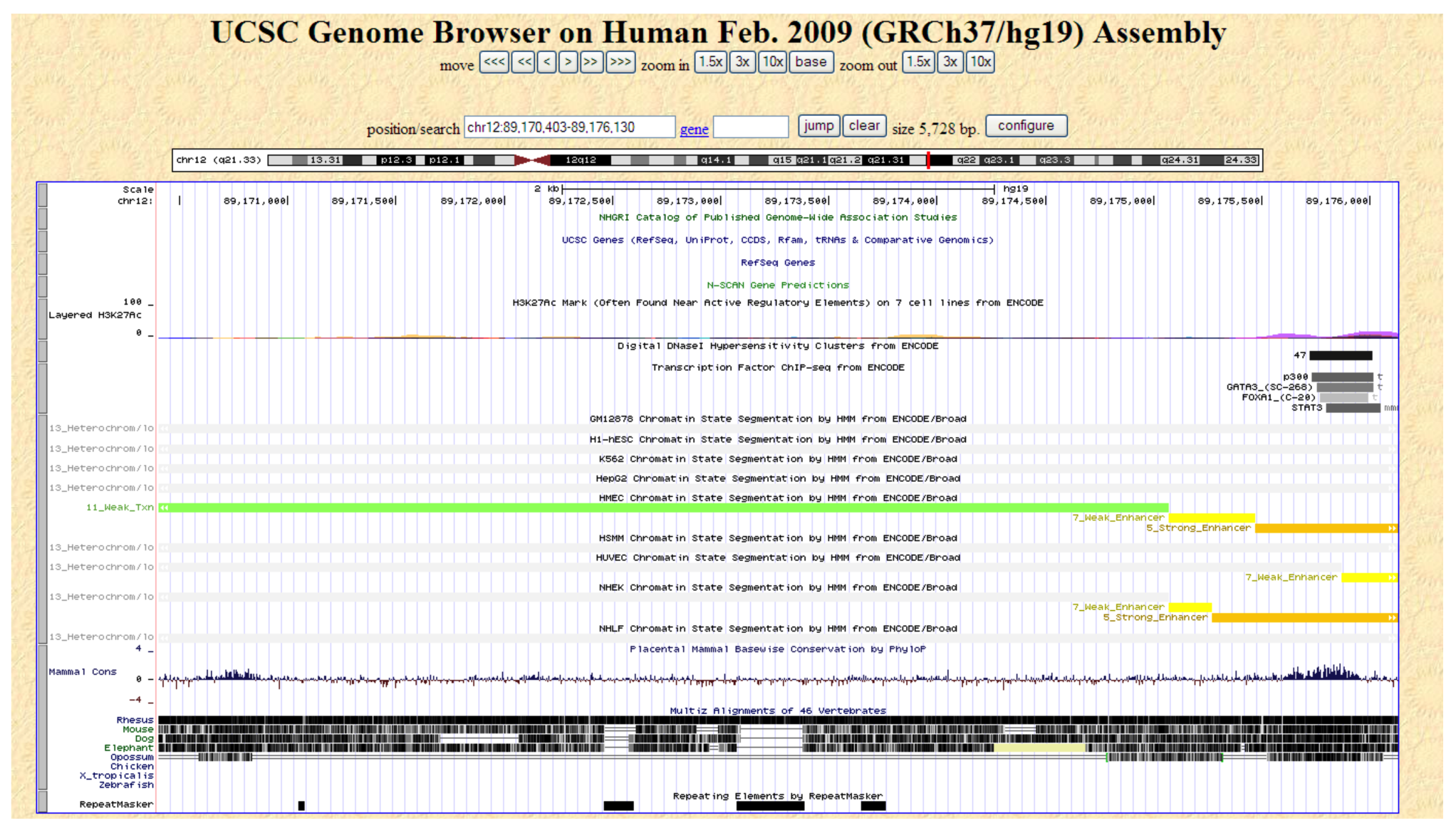

Supplement: Figure S3 — The human genome sequence that corresponds to the SCCD 5.7 Kb CNV contains enhancer element signatures. The UCSC Genome Browser display is of human chromosome 12 from 89,170,403 to 89,176,159 (build GRCh37). Multiple tracks are presented including the ENCODE DNaseI Hypersensitivity, ENCODE ChIP-seq and ENCODE/Broad Chromat in State Segmentation tracks. (TIF) [file pgen.1003409.s003.tif]
